# Application of the hybrid BOPPPS teaching model in clinical internships in gynecology

**DOI:** 10.1186/s12909-023-04455-2

**Published:** 2023-06-22

**Authors:** Zhengfen Xu, Xuan Che, Xiaodi Yang, Xiaoxia Wang

**Affiliations:** 1 Department of Science and Education, Jiaxing Maternity and Child Health Care Hospital, College of Medicine, Jiaxing University, Jiaxing, 314000 China; 2 Department of gynecology, Jiaxing Maternity and Child Health Care Hospital, College of Medicine, Jiaxing University, Jiaxing, 314000 China

**Keywords:** BOPPPS, hS, Ybrid teaching model, Clinical internship, Gynecology

## Abstract

**Background:**

The traditional gynecological teaching model is not conducive to the cultivation of trainee doctors’ clinical skills, thinking patterns and doctor‒patient communication ability. This study aims to explore the effect of the application of the hybrid BOPPPS (bridge-in, objective, preassessment, participant learning, postassessment, summary) teaching model in clinical internships in gynecology.

**Methods:**

This observational study was conducted among final-year undergraduate medical trainee doctors at Jiaxing Maternity and Child Health Care Hospital from September 2020 to June 2022. Members of the control group were introduced to the traditional teaching model, while members of the experimental group were introduced to the hybrid BOPPPS teaching model. Trainee doctors’ final examination scores and teaching satisfaction were compared.

**Results:**

The control group consisted of 114 students who entered the university to pursue undergraduate degrees in 2017, and the experimental group consisted of 121 students who entered the university to pursue undergraduate degrees in 2018. The final examination scores attained by trainee doctors in the experimental group were higher than those attained by trainee doctors in the control group (P < 0.05). The final theoretical exam scores attained by members of the control group were significantly higher than their preassessment scores (P < 0.01). The scores differed significantly between female and male subjects before the internship (p<0.05) but not after the internship (p>0.05). In total, 93.4% of trainee doctors in the experimental group thought that the hybrid BOPPPS teaching model helped them improve their case analysis ability, and the difference in this measure between the experimental and control groups was statistically significant (P < 0.05). A total of 89.3% of trainee doctors in the experimental group supported the promotion and application of the hybrid BOPPPS model in practice in other disciplines.

**Conclusion:**

The hybrid BOPPPS teaching model helps improve trainee doctors’ learning environment, stimulate their interest and initiative in learning, enhance their clinical practice ability and increase their satisfaction; therefore, this model is worth promoting and applying in practice in other disciplines.

**Supplementary Information:**

The online version contains supplementary material available at 10.1186/s12909-023-04455-2.

## Background

Clinical internships represent an important stage for medical undergraduates, enabling them to integrate theoretical knowledge with clinical skills and form their own clinical thinking patterns for the first time [1]. In the traditional gynecological teaching model in China, clinical teachers are very busy and focus on patients, and trainee doctors are mainly observers. Interactions between clinical teachers and trainee doctors are insufficient and inefficient [2]. Moreover, since gynecological diseases often require a focus on patients’ privacy, trainee doctors, especially male trainees, always face restrictions when observing treatment or performing gynecological examinations [3]. Therefore, trainee doctors have few opportunities to practice. The internship process may be boring for trainee doctors, and trainee doctors lack motivation for active learning, which is not conducive to the cultivation of clinical skills, thinking patterns and doctor‒patient communication ability [4]. For the reasons above, we tried to change the traditional teaching model in China to cultivate trainee doctors’ ability to analyze and solve problems and to cultivate modern high-quality doctors.

The BOPPPS teaching model was introduced as part of teaching reform at our institution to stimulate trainee doctors’ learning initiative and take full advantage of teachers’ guidance. To reduce the extent to which gynecologists were busy with clinical work, we introduced a teaching post in which senior gynecologist attending physicians took time off from clinical work in turns for one year to serve as full-time teaching administrator. In this way, they could receive credits toward promotion.

The BOPPPS was first proposed by training institutions of universities in North America to increase teaching skills [5]; the model emphasizes all-round participation and interactive feedback between teachers and trainee doctors with a primary focus on the trainee doctors themselves. We integrated the BOPPPS model with an online teaching platform to promote the utilization of teaching resources and the development of teaching interaction, to enable trainee doctors to better participate in the whole process of learning activities and to improve their enthusiasm and initiative for learning [6–8]. In September 2021, we implemented the hybrid BOPPPS teaching model in gynecology. The process and results are reported as follows.

## Methods

### Participants

This observational study was conducted among final-year undergraduate medical trainee doctors from September 2020 to June 2022 at Jiaxing Maternity and Child Health Care Hospital. All trainee doctors provided informed consent regarding the study content.

### Design

This study used a trial-controlled method in accordance with the ethical principles of medical research in the Declaration of Helsinki. The control group consisted of students who entered the university to pursue undergraduate degrees in 2017, who took turns completing internships in the gynecology department from September 2020 to June 2021. These trainee doctors received the traditional teaching model. The experimental group consisted of students who entered the university to pursue undergraduate degrees in 2018, who took turns completing internships in the gynecology department from September 2021 to June 2022. These trainee doctors were introduced to the hybrid BOPPPS teaching model. Both groups of students completed their internships in the last year (fifth year) of their undergraduate studies.

### Recruitment of patients

To encourage the participation of patients, we provided specific instructions to patients who were suitable for the new teaching model. The instructions emphasized that trainee doctors would provide them with a detailed and comprehensive medical history inquiry and physical examination under the guidance of a senior attending physician.

### Teaching methods

Trainee doctors in the control group received the traditional teaching model: clinical teachers from different gynecological wards were assigned to the trainee doctors, and the teachers guided the trainee doctors in observing the clinical process in the outpatient department, inpatient department and operation room.

**Fig. 1:**
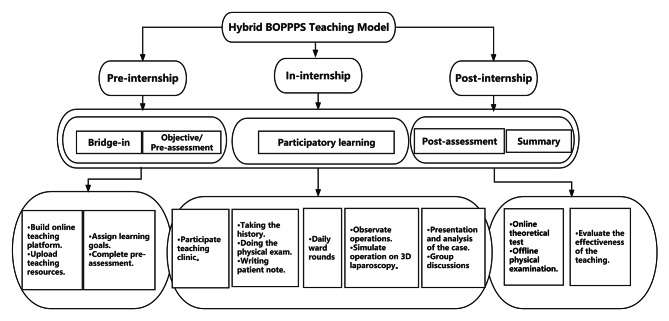
The specific process of the hybrid BOPPPS teaching model

Every two weeks, approximately 10 trainee doctors in the experimental group came to the gynecology department for their rotation. We divided the trainee doctors into five groups, with two people in each group, and each group was assigned to a different ward. Trainee doctors received the hybrid BOPPPS teaching model under the guidance of the teaching administrator. The specific process was as follows (Fig. 1).

### Hybrid BOPPPS teaching model

1. **Bridge-in**: Before the internship, we built an online teaching platform and uploaded teaching resources, including 5 typical representative cases of gynecological common conditions (vaginal infections, uterine fibroids, ovarian cysts, ectopic pregnancy and abortion), relevant theoretical knowledge, the latest literature, and videos and pictures of clinical operations.
2. **Objective**: According to the syllabus, we listed the specific requirements of theoretical knowledge and clinical skills for each case, such as mastery of gynecological physical examination and definition of ectopic pregnancy.
3. **Preassessment**: After assigning the learning goals, we gave trainee doctors one week for self-study and then organized an online theoretical test that tested case analysis ability before the internship. The teaching administrator analyzed the test results and identified the knowledge points with a high error frequency for the clinical teacher to emphasize these knowledge points during the internship.
4. **Participatory learning**: Trainee doctors rotated in the gynecology department for 2 weeks, including five days in wards, three days in the abortion operation room and two days in the outpatient department. Trainee doctors were randomly divided into 5 groups, and each group was assigned a condition mentioned above. In the outpatient clinic, trainee doctors were required to participate in the teaching clinic and record 20 case diagnoses, auxiliary examinations and diagnosis plans at least every day, and after receiving feedback from the teacher, they had to upload the record to the teaching platform as part of their daily performance. In the gynecological ward, trainee doctors were required to manage patients under their teacher’s guidance and supervision, and they were also required to prepare a PPT for presentation based on the assigned case. The teacher organized a trainee doctor-led case report every week in which they required trainee doctors to fully explore relevant basic and clinical problems from real clinical cases and guided them in connecting relevant knowledge points.
5. **Postassessment**: Postassessment was organized on the last day of the rotation, and it included an online theoretical test and an offline clinical skill test. The theoretical test also tested case analysis ability, such as the preassessment, and the difficulty coefficient was the same.
6. **Summary**: Clinical teachers answered any questions trainee doctors asked, summarized the whole internship process, reinforced the key and difficult points, and expanded upon the teaching content.

### Questionnaire survey

Before the end of the internship, trainee doctors were required to complete an anonymous questionnaire on the online platform to assess their satisfaction with teaching. The questionnaire was designed by the study team and comprised 9 questions concerning whether the learning objectives were clear, whether case analysis ability was improved, and whether clinical skills were improved. For trainee doctors in the experimental group, another question was added regarding whether they supported the promotion of the new teaching model.

### Statistical analysis

Data were analyzed in IBM SPSS Statistics 25 (IBM, New York, USA). We used the Kolmogorov-Smirnov test to evaluate the normality of quantitative variables. Metrological data were reported as the mean ± standard deviation (SD) and compared between groups using the independent sample t test. Classification data were reported as numbers and proportions (%) and compared between groups by analysis of variance. P < 0.05 was considered statistically significant.

## Results

### Comparison of general information between the two groups

A total of 235 trainee doctors participated in the study: 125 (51.5%) were in the experimental group, and 114 (48.5%) were in the control group. Trainee doctors in the experimental group consisted of 65 males and 59 females with a mean age of 22.50 ± 0.51 years. The control group consisted of 56 males and 55 females with a mean age of 22.46 ± 0.5 years. The mean final examination score in the last semester was 84.84 ± 5.47 in the experimental group and 84.98 ± 5.69 in the control group. There was no significant difference in terms of general information between the two groups (all P > 0.05). (Table 1).

**Table 1:** Comparison of general information between the two groups

Variable	Experimental group (n = 121)	Control group (n = 114)	χ^2^/t	P value
Gender n(%)				
Male	65(53.72)	59(51.75)	0.091	0.763
Female	56(46.28)	55(48.25)		
Age (year)	22.50 ± 0.51	22.46 ± 0.5	0.473	0.451
Final examination score of last semester	84.84 ± 5.47	84.98 ± 5.69	0.203	0.694

### Comparison of the postassessment scores of the two groups

For all subjects (p<0.01), female subjects (p<0.01) and male subjects (p<0.05), the mean theoretical knowledge, clinical skills, and total scores attained by members of the the experimental group were significantly higher than those attained by members of the control group. (Table 2).

**Table 2:** Comparison of the postassessment scores of the two groups

Group	Variable	Experimental group (n = 121)	Control group (n = 114)	t	p
Total subjects					
	Theoretical knowledge	84.45 ± 6.21	81.04 ± 7.54	-4.01	<0.001
	Clinical skill	91.30 ± 3.75	88.93 ± 5.21	-3.78	<0.001
	Total	87.87 ± 4.73	84.99 ± 5.69	-4.23	<0.001
Female subjects					
	Theoretical knowledge	84.68 ± 6.49	81.73 ± 6.21	-3.78	<0.001
	Clinical skill	91.88 ± 3.09	89.22 ± 5.22	-3.27	<0.001
	Total	88.81 ± 3.90	85.47 ± 5.17	-3.85	<0.001
Male subjects					
	Theoretical knowledge	84.43 ± 6.00	80.41 ± 8.59	-2.08	0.039
	Clinical skill	90.80 ± 4.20	88.66 ± 5.23	-2.52	0.013
	Total	87.06 ± 5.25	84.53 ± 6.15	-2.47	0.015

### Comparison of the theoretical knowledge scores before and after the internship in the experimental group

In the experimental group, for all subjects, female and male subjects, their mean theoretical knowledge scores after the internship were significantly higher than those before the internship (p<0.01). The scores differed significantly between female and male subjects before the internship (p<0.05) but not after the internship (p>0.05). (Table 3)

**Table 3:** Comparison of the theoretical knowledge scores before and after the internship in the experimental group

Group	Before internship	After internship	t	p
Total subjects	72.26 ± 9.77	84.45 ± 6.21	-10.51	<0.001
Female subjects	75.52 ± 7.22^a^	84.68 ± 6.49	-7.06	<0.001
Male subjects	71.80 ± 11.20^b^	84.43 ± 6.00	-8.01	<0.001

The difference between a and b is statistically significant (p<0.05)

### Comparison of satisfaction between the two groups

In total, 93.4% of trainee doctors in the experimental group believed that their case analysis ability had improved after the internship; this proportion was 79.8% in the control group, and the difference was statistically significant (P = 0.002). A total of 88.4% of trainee doctors in the experimental group believed that the teaching model improved their self-study ability, while this proportion was only 65.8% in the control group; the difference showed statistical significance (P = 0.001). A total of 89.3% of trainee doctors in the experimental group supported the promotion of the hybrid BOPPPS teaching model. (Table 4).

**Table 4:** Comparison of satisfaction between the two groups

Evaluation content	Experimental group (n = 121)	Control group (n = 114)	χ^2^	P
Learning goals are clear	104(86%)	81(71.1%)	7.778	0.005
Contributes to the understanding of theoretical knowledge	87(71.9%)	59(51.8%)	10.125	0.001
Improve case analysis ability	113(93.4%)	91(79.8%)	9.430	0.002
Improve literature review ability	77(63.6%)	56(49.1%)	5.033	0.025
Cultivate self-study ability	107(88.4%)	81(65.8%)	11.078	0.001
High participation in discussion	79(65.3%)	65(57%)	1.693	0.193
Stimulate the interest in learning	105(86.8%)	78(68.4%)	11.478	0.001
Improve teamwork ability	73(60.3%)	55(48.2%)	3.457	0.063
Study pressure is high	97(80.2%)	73(64%)	7.633	0.006
Support the promotion of hybrid BOPPPS teaching models	108(89.3%)			

## Discussion

### The hybrid BOPPPS teaching model helps improve trainee doctors’ learning environment

In the traditional gynecological teaching model in China, clinical teachers are very busy and always inadequately prepared for internships; thus, they occasionally merely duplicate previous instances of classroom instruction. In this situation, trainee doctors are mainly observers and have little practical experience, and interactions between clinical teachers and trainee doctors are insufficient and inefficient. Poor teaching quality urgently needs improvement; thus, we introduced a teaching post in which senior gynecologist attending physicians took turns setting aside their clinical work for one year to serve as full-time teaching administrator. In this way, teaching administrators could receive credits toward promotion, thus motivating them. In addition, the administrates thus had sufficient time to participate in the latest training in educational practice, implement internship programs, collect typical representative cases, communicate adequately with trainee doctors, strengthen their teaching management skills, implement teaching reform and improve their teaching quality. In our study, 86% of trainee doctors in the experimental group thought the learning goals were clear; however, only 71.1% of trainee doctors in the control group expressed the same opinion, and the difference was statistically significant (P<0.01). Previous studies have shown that full-time teachers are likely to be acquainted with developments in curriculum and changes in pedagogy pertaining to their subject [9].

In the traditional teaching model in China, women often refuse to allow trainee doctors, especially male trainees, to observe their treatment or gynecological examinations because the process may violate their privacy, which would cause them to feel embarrassed and uncomfortable. To encourage participation from patients, we provided specific instructions to patients who were suitable for the new teaching model. The instructions emphasized the fact that trainee doctors would provide them with a detailed and comprehensive medical history inquiry and physical examination under the guidance of a senior attending physician. This approach contributed to the establishment of better doctor‒patient relationships and the promotion of cooperation. The learners also reported that effective communication with patients and the offer to have a support person present before the gynecological examination and treatment could reduce women’s anxiety and fear and thus promote their cooperation [10].

### The hybrid BOPPPS teaching model is helpful for improving trainee doctors’ interest and initiative in learning

The situation that patients refuse examination and treatment, or even observation, by trainee doctors, especially male trainee doctors imposes significant psychological distress on them, who suffer from a lack of clinical opportunity to practice [11]. When we used the hybrid BOPPPS teaching model, we explained the program to the patients in advance, so most of the patients cooperated with the trainee doctors, including the male students; thus, trainee doctors had more opportunities to perform gynecological examinations on real patients. This approach is helpful for improving trainee doctors’ interest and initiative in learning, especially for male students. In our study, we found that the scores differed significantly between female and male subjects before the internship but not after the internship. This finding may be due to the fact that the male trainee doctors were not interested in gynecology before the internship, and the new model internship changed their opinion in this respect.

The new teaching model emphasizes intern-centered, all-round participation, which provides more opportunities for trainee doctors to practice [2, 7]. Before the internship, we uploaded relevant resources to the online teaching platform, including typical representative cases of gynecological common conditions, the latest relevant literature, and videos and pictures of clinical operations, and we listed the specific requirements of the course, which was designed as a self-learning resource for trainee doctors. With this knowledge, trainee doctors had greater self-confidence when they faced patients, which led to better treatment options and diagnostics. Previous studies have shown that case-based learning could play a supportive role in medical student internships [12, 13]. During internships, trainee doctors must manage patients independently and prepare to present cases, so they must be fully engaged. In our study, 86.8% of trainee doctors in the experimental group thought the hybrid BOPPPS helped stimulate their interest in learning, and 88.4% thought the model helped cultivate their self-study ability; this finding is consistent with the result of a past study [7]. Therefore, the intern-centered teaching model was conducive to the cultivation of trainee doctors’ active thinking ability.

### The hybrid BOPPPS teaching model helps improve trainee doctors’ clinical practice ability

One of the objectives of internships is to improve trainee doctors’ ability to diagnose and treat common and frequently occurring gynecological diseases [14]. In the bridge-in process, we listed five typical representative cases of common gynecological common conditions (vaginal infections, uterine fibroids, ovarian cysts, ectopic pregnancy and abortion) for trainee doctors to self-study to consolidate the relevant theoretical knowledge. In this way, trainee doctors can improve their clinical diagnostic and therapeutic capabilities in clinical practice [15]. In the process of participatory learning, we helped trainee doctors integrate theoretical knowledge with “real” clinical cases. They took patients’ histories, performed gynecological examinations, made efficient clinical decisions and created case reports under the teacher’s supervision, which helped improve their clinical thinking and self-reflection ability. Our study showed that 93.4% of trainee doctors in the experimental group believed that their case analysis ability had improved. For members of the experimental group, their mean theoretical knowledge score before the internship was significantly higher than their score after the internship. Although the mean final examination scores of the experimental group were higher than those of the control group, the test scores did not differ substantially between groups. This finding may be due to the wide range of our examinations and the students may have simply spent more energy on the five typical gynecological cases. In the future, we will list more cases and promote and apply the new model in practice in other disciplines in our hospital to ensure that trainee doctors can master more knowledge and skills.

### The hybrid BOPPPS teaching model helps improve trainee doctors’ satisfaction

The hybrid BOPPPS teaching model uses a multimedia approach in the internship process, which contributes to an active learning atmosphere and improves study comprehension. Previous studies have shown that the use of multimedia can improve students’ learning motivation and strengthen their practical ability [16, 17]. In this study, trainee doctors were at liberty to view the teaching resources, share their experiences and ask questions on the platform. In real clinical settings, trainee doctors experience responsibility and pressure, and they also have the motivation to manage every patient well. If patients recover well, trainee doctors’ confidence increases. This is very important for them to become competent doctors in the future. The new teaching model was carried out with the help of the teaching administrator, which reduced the pressure on clinical teachers. Most gynecologists are very busy, and teaching is a part of their work [18]. Therefore, we selected a full-time teaching administrator who enjoys teaching and interacting with trainee doctors. This gives trainee doctors the opportunity to communicate and interact directly with the administrator. Previous studies have shown that students perceive feedback from supervisors as facilitating their growth [19, 20]. In our study, no trainee doctors withdrew from the study, and 89.3% of trainee doctors in the experimental group supported the promotion of the hybrid BOPPPS teaching model. This is consistent with previous research [2].

Several limitations also exist in our study, and much remains to be improved. First, our study showed that 80.2% of trainee doctors in the experimental group thought their learning pressure was high. In the traditional teaching model, trainee doctors only need to observe their teachers’ procedures and listen to their teachers’ explanations [21]. However, in the new teaching model, we proposed higher requirements for trainee doctors. They had to finish self-study before the internship, and they had to participate in the teaching clinic, manage patients independently and complete case reports. Trainee doctors have become accustomed to the traditional teaching model and cannot adapt to the new model immediately. In the future, we will focus on developing approaches for trainee doctors to become more self-motivated. Second, the new teaching model poses new challenges for clinical teachers. In addition to completing daily teaching, they must search the literature, record videos and communicate with trainee doctors. Of course, this also plays a positive role in promoting their teaching ability [22]. In addition, we did not perform a statistical analysis of patients’ acceptance of the possibility of trainee doctors taking a full history and performing an examination in the new teaching model as compared to the traditional model, although this information would have been a valuable consideration. However, our observations suggest that the acceptance rate increased significantly compared to that associated with the traditional teaching model. For female subjects, the acceptance rate improved from approximately 50–95%, while for male subjects, the acceptance rate improved from approximately 20–90%. If we had data regarding the specific acceptance rates, we may have been able to conclude that patients play a significant role in influencing the effectiveness of internships. In the future, we will conduct relevant surveys to study the factors that influence women’s acceptance of the possibility of allowing trainee doctors to take a full medical history and perform gynecological examinations. In addition, in line with the factors observed, corresponding reform projects should be developed. For example, social publicity should be strengthened: teaching is an important task of teaching hospitals, and medicine is a practical science. To incorporate advanced medical technology, the active cooperation of patients is urgently necessary. Therefore, we encourage women to cooperate actively with clinical teaching work and clinical teaching. Thus, trainee doctors can successfully complete the gynecology internship tasks, laying a solid foundation for their future work and learning.

## Conclusion

The intern-centered hybrid BOPPPS teaching model helps improve trainee doctors’ learning environment, stimulate trainee doctors’ interest and initiative in learning, enhance their self-study ability, and improve their clinical skills. The overall satisfaction level with the new teaching model was found to be high in our study, and trainee doctors’ attitudes were quite positive; most of them supported the promotion and application of the hybrid BOPPPS in practice in other disciplines.

## Electronic supplementary material

Below is the link to the electronic supplementary material.

Supplementary Material 1

## Data Availability

All data generated or analyzed during this study are included in this published article. The SPSS raw dataset can be provided on request. The corresponding author Xiaoxia Wang will provide additional data, if requested.

## Declarations

All procedures performed in studies involving human participants were in accordance with the ethical standards of the Institutional and/or National Research Committee and with the 1964 Helsinki Declaration and its later amendments, or comparable ethical standards. The study was approved by the ethics committee of Jiaxing Maternity and Child Health Care Hospital, College of Medicine, Jiaxing University and all the participants signed the informed consent.

## Abbreviations

BOPPPS: bridge?in, learning objective, pretest, participatory learning, posttest, and summary
