# Supplementary material for: Application of the hybrid BOPPPS teaching model in clinical internships in gynecology
Source: BMC Med Educ. 2023 Jun 22;23:465. doi: 10.1186/s12909-023-04455-2 (PMC10286474; doi:10.1186/s12909-023-04455-2)
Supplement: Supplementary file 1 — Supplementary Material 1 [file 12909_2023_4455_MOESM1_ESM.pdf]

Jiaxing Maternity and Child Health Care Hospital

Instructions for Patients

Dear patient friend:

I am very glad that you can participate in our gynecology teaching reform program. In this program the trainee doctors will provide you with a detailed and comprehensive medical history inquiry and physical examination under the guidance of a senior attending physician. This teaching reform can improve the knowledge and experience of trainee doctors, and will provide great help for them to face the real clinical environment in the future. Your support is a contribution to the development of future excellent doctors!

Thank you for your cooperation!
